# Mean platelet volume in young children with urinary tract infection

**DOI:** 10.1038/srep18072

**Published:** 2015-12-15

**Authors:** I Re Lee, Jae Il Shin, Se Jin Park, Ji Young Oh, Ji Hong Kim

**Affiliations:** 1 Department of Pediatrics, Yonsei University College of Medicine, Seoul, Korea; 2 Department of Pediatric Nephrology, Severance Children's Hospital, Seoul, Korea; 3 Department of Pediatrics, Ajou University School of Medicine, Daewoo General Hospital, Geoje, Korea

## Abstract

Mean platelet volume (MPV) has not yet been well-established in urinary tract infection (UTI). The purpose of this study was to evaluate the role of MPV as an acute phase reactant in children with UTI. Data from 118 young children (<2 years) with UTI between 2012 and 2013 were grouped as acute pyelonephritis (APN) and lower UTI according to the dimercaptosuccinic acid (DMSA) scan abnormalities. MPV, platelet distribution width (PDW) platelet count, and other infection markers (white blood cell [WBC] count, erythrocyte sedimentation rate [ESR], and C-reactive protein [CRP]) were measured. WBC (*P* = 0.001), ESR (*P* = 0.005), CRP (*P* < 0.001) and MPV levels (*P* = 0.011) were significantly higher in the APN group than those in the lower UTI group. MPV positively correlated with PDW, CRP and negatively with platelet count. Multiple logistic regression analyses showed that CRP and MPV were independent predictive factors for APN patients. However, the area under the Receiver Operating Characteristic (ROC) curve analysis for MPV was lower than CRP. Our results suggest that MPV can be an inflammatory marker in UTI, but the predictive value of MPV was not superior to CRP in the diagnosis of APN.

Urinary tract infection (UTI) is one of the common bacterial infections in children and may lead to serious morbidity and mortality^1^,^2^. UTI associated with high grade vesicoureteral reflux (VUR) can lead to renal scarring and chronic renal failure^3^. Clinical symptoms and some inflammatory markers have been used for the differentiation of upper from lower UTI^4^,^5^. Laboratory findings of UTI usually show leukocytosis, neutrophilia, and increased erythrocyte sedimentation rate (ESR) or C-reactive protein (CRP) levels^4^,^5^.

Dimercaptosuccinic acid (DMSA) scan is used in distinguishing acute pyelonephritis (APN) from lower UTI, but it can expose the patients to radiation^6^,^7^. Platelets play an important role in the pathogenesis of various infectious or inflammatory disorders^8^,^9^. Platelet counts and mean platelet volume (MPV) have been studied as inflammatory markers in relation to the disease activity^10^. MPV may be increased in mild inflammation due to the emergence of the large platelets in the peripheral circulation and conversely, may be decreased in severe inflammation because of the consumption of large platelets in the inflammatory area^11^,^12^. MPV has also been studied as an inflammatory marker in various infectious disorders, not only as a negative marker with rotavirus gastroenteritis, but also as a positive marker with hepatitis B, acute appendicitis, and sepsis^12^,^13^,^14^,^15^.

In this study, we compared MPV in children with APN and with low UTI to determine whether it could be used as an inflammatory marker in distinguishing APN with lower UTI.

## Results

Clinical characteristics and laboratory findings of the patients were presented in Table 1. There were no significant differences regarding the age at presentation and gender between the APN and lower UTI groups. WBC counts were significantly higher in the APN group than those in the lower UTI group (*P* = 0.001). ESR (*P* = 0.005) and CRP levels (*P* < 0.001) were also significantly higher in the APN group than those in the lower UTI group. The incidence of VUR was more frequent in the APN group (*P* = 0.004), but abnormal USG findings did not differ between the two groups. There were significant differences between the APN and lower UTI group in terms of MPV levels (*P* = 0.011), while platelet count (*P* = 0.742) and PDW (*P* = 0.451) did not differ between the two groups.

**Figure 1:**
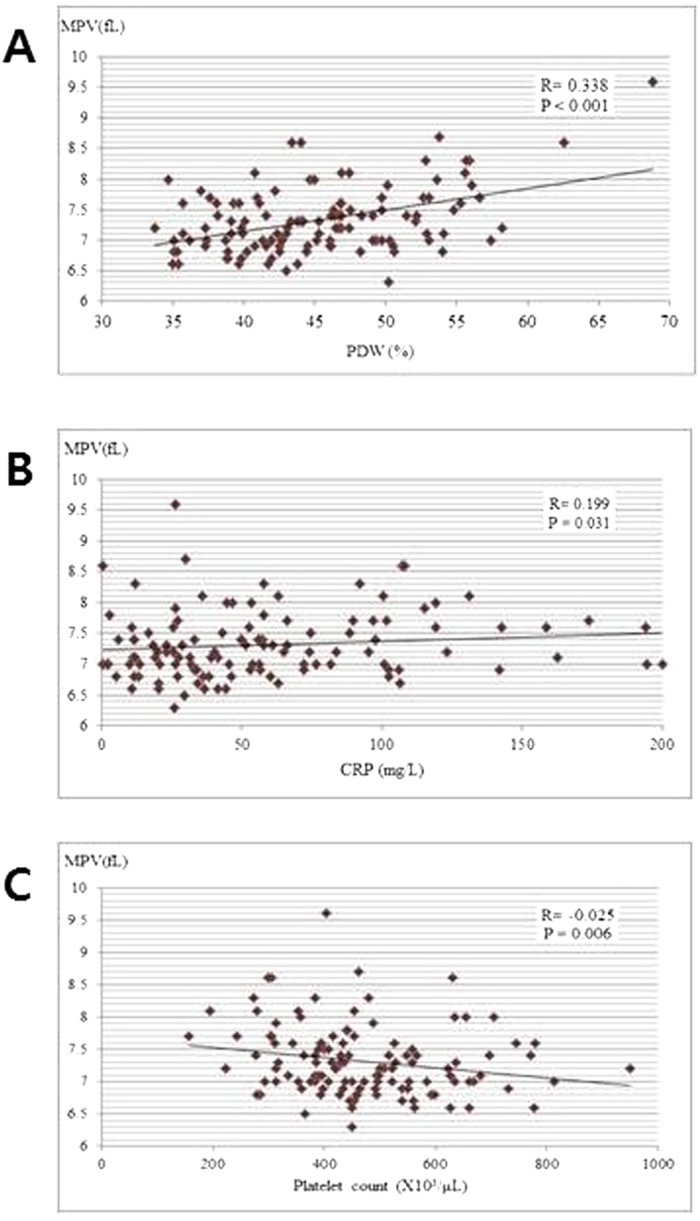
Correlation among mean platelet volume (MPV), (A) platelet distribution width (PDW), (B) C-reactive protein (CRP), and (C) platelet count in UTI patients.

Moreover, MPV positively correlated with PDW (*R* = 0.338, *P* < 0.001), CRP levels (*R* = 0.199, *P* = 0.031) and negatively with platelet count (*R* = −0.025, *P* = 0.006) (Fig. 1). Multiple logistic regression analyses showed that CRP (odds ratio [*OR*]: 1.026, 95% *CI* : 1.013–1.040, *P* < 0.001), and MPV levels (*OR*: 2.393, 95% *CI*: 1.019–5.620, *P* = 0.045] were independent predictive factors for positive DMSA defects in UTI patients (Table 2). However, MPV levels were not different according to gender (male 7.4 ± 0.6 vs. female 7.5 ± 0.5 fL in the APN group [*P* = 0.878] and male 7.2 ± 0.5 vs. female 7.2 ± 0.6 fL in the lower UTI group [*P* = 0.927]) and those were not correlated with age (*R* = −0.092, *P* = 0.478 in the APN group and *R* = −0.217, *P* = 0.107 in the lower UTI group) or the duration of fever (*R* = −0.036, *P* = 0.701).

**Figure 2:**
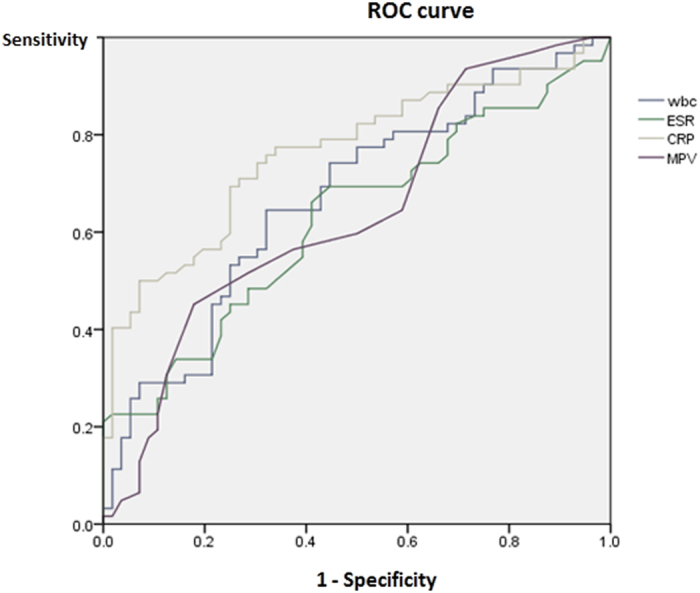
Receiver operating characteristic curve of mean platelet volume (MPV) and other inflammatory markers for APN.

We also analyzed the diagnostic properties of the various inflammatory markers, using ROC curves (Fig. 2). Area under the curves (AUC) values from ROC curve analysis for WBC, ESR, CRP, and MPV were 0.670 (*P* = 0.001, 95% *CI*: 0.573–0.768), 0.626 (*P* = 0.018, 95% *CI*: 0.526–0.727), 0.764 (*P* < 0.001, 95% CI: 0.678–0.850), and 0.641 (*P* = 0.008, 95% CI: 0.541–0.742), respectively (Table 3). The AUC values for CRP were higher than those of other inflammatory markers such as WBC, ESR, and MPV levels. The AUC values for MPV were lower than those of CRP, but higher than those of ESR.

The sensitivity, specificity, positive and negative predictive values, positive and negative likelihood ratios of the MPV were determined with different cut-off values, which were compared with a previous study (Table 4). MPV, the cut-off value of 7.4 fL, and with AUC of 0.641, detected APN patients among those with UTI. The sensitivities and specificities for MPV were found to be 45.2% and 82.1%, respectively.

We found that MPV levels were significantly increased one day after antibiotic therapy (*P* = 0.031) and were similar 2–5 days after antibiotic therapy and were significantly decreased >6 days after antibiotic therapy (*P* *=* 0.046) compared with initial levels (Table 5).

## Discussion

The early diagnosis of APN may be difficult during infancy, but differentiation of APN from lower UTI is important due to renal parenchymal damage, which can cause renal scarring that may lead to renal hypertension and chronic renal failure^2^,^3^. To differentiate APN from lower UTI, DMSA scan can be used, but it can expose the patients to radiation and require sedation in young infants and children^16^,^17^,^18^.

There have been a few reports which have evaluated the relationship between MPV and infectious disorders^12^,^13^,^14^,^15^. Some studies suggested that MPV increased as a positive acute phase reactant^13^,^14^, while others reported that it decreased as a negative acute phase reactant^12^. MPV as a positive acute phase reactant has much been studied in patients with sepsis. Aydemir *et al.* reported that MPV was significantly increased for the first 3 days of patients with Gram-positive sepsis, for 4 days in Gram-negative septic patients, and for all 5 days in fungal septic patients (*P* < 0.001)^15^. Guida *et al.* also showed that sepsis was frequently associated with thrombocytopenia and an elevation in MPV in very low birth weight infants^19^. Icli *et al.* also demonstrated that MPV levels were higher in patients with infective endocarditis and decreased significantly after treatment^20^. Increased MPV may indicate an increased proportion of young platelets in the peripheral circulation, and is suggestive of increased platelet production or increased platelet destruction^21^. Currently, however, there is no study how fast MPV is elevated in response to infection, requiring future studies.

Conversely, Albayrak *et al.* reported significantly lower MPV in adults who sustained acute appendicitis than in controls^14^. The reason for the reduced MPV in acute appendicitis is unclear, but the reduced MPV could be due to the consumption or sequestration of the large activated platelets in intestinal vasculature^22^. In addition to sepsis and various infections, MPV also has a positive correlation with many different diseases such as diabetes, myocardial infarction and young children with infections and varying degrees of the cut-off value (8.0–10.35 fL), sensitivity (53–98%) and specificity (35–87%)^23^,^24^,^25^,^26^,^27^.

Regarding the role of MPV in UTI, Catal *et al.* reported an increase in MPV in children with APN compared to normal children^28^. However, they did not analyze MPV levels between APN and lower UTI according to the DMSA scan abnormalities and used the bag urine in some patients as a collection method in the diagnosis of UTI, which might lead to contamination. Tekin *et al.* showed that the sensitivity and specificity of the MPV using a cut-off value of 8.2 fL was 81.4% and 86.3%, respectively, in predicting APN which were higher than those of the WBC, CRP, and ESR levels^29^.

In the present study, we found increased levels of MPV in children with APN than those with lower UTI, suggesting MPV acted as a positive acute phase reactant in UTI. We also showed that MPV levels had a positive correlation with CRP levels, which further supports previous findings^28^,^29^. However, the sensitivity and specificity of the MPV using a cut-off value of 7.4 fL was 45.2% and 82.1%, respectively, in predicting APN and the sensitivity of MPV in predicting APN was lower than that of Tekin *et al.*’s study^29^. One of the differences between Tekin *et al.*’s study and ours is the age of the patient groups^29^. Tekin *et al.*’s study included the patients with a broad range of age from 2 months to 12 years while we did young infants with less than 2 years of age^29^. Therefore, MPV may be an adjunctive inflammatory marker rather than an absolute biomarker for APN in UTI, considering the predictive value of MPV was not superior to CRP in the diagnosis of APN. In addition, platelet count and PDW did not differ between children with APN and those with lower UTI in our study.

Catal *et al.* speculated that the rise in MPV in APN can be caused by an increased production of larger and/or younger platelets as a reaction to infection-related platelet destruction, and increased levels of β-thromboglobulin in bacterial infections might activate and release of platelets^30^. However, we would like to add another pathomechanism. Although not studied in UTI, there have been some reports on the effect of thrombopoietin (TPO) on MPV levels, and Senaran *et al.* reported that there was a positive correlation between TPO levels and MPV values (*P* < 0.05) in patients with coronary artery disease^31^. TPO is thought to be the primary physiological regulator of megakaryopoiesis, and injection of recombinant TPO protein selectively induced thrombocytosis *in vivo*^32^. Also, cytokines such as interleukin (IL)-6, erythropoietin, stem cell factor, and granulocyte–macrophage–CSF are known to play a role in megakaryopoiesis^33^,^34^. Because an inflammatory cytokine, IL-6, increased in APN and is associated with renal scarring^35^, there is a possibility that MPV might be increased due to increased TPO production induced by IL-6 in APN.

Our study has some limitations: the number of patients was not many; the values of normal young children have not been established yet. Although MPV levels did not correlate with the duration of fever in our study, there is the possibility that the time might have an important impact on the MPV levels. We used EDTA as an anticoagulant and analyzed MPV levels within 1 hour, but those levels might be affected by the use of anticoagulants^36^.

In conclusion, we found that MPV acted as a positive acute phase reactant in children with UTI, and MPV levels were significantly elevated in children with APN on DMSA scan. However, the predictive value of MPV was low in the diagnosis of APN. Nevertheless, our results may contribute to our understanding of MPV as an inflammatory marker in UTI.

Further studies are necessary to evaluate the relationship among MPV levels, TPO, and IL-6 levels in UTI to elucidate the kinetics of megakaryopoiesis in response to various stimuli in the future, although we did not measure these parameters.

## Methods

We retrospectively analyzed the data from 118 children (86 boys and 32 girls, aged 2–24 months; mean age of 4.8 ± 3.4 months) with febrile UTI admitted to Severance Children’s Hospital from January 2012 to December 2013. Fever was defined as a body temperature ≥38 °C^37^. All patients were diagnosed with UTI who had culture growing >50,000 colonies of one single bacterial species on a urine sample obtained by catheterization^38^. We included only patients who had received blood sampling before administration of antibiotics, because the concomitant antibiotic therapy can affect the MPV levels. We also analyzed the data according to the age and gender to elucidate whether MPV levels are affected by these parameters.

Clinical data such as age, sex, and duration of fever were recorded. Before initiation of antibiotic treatment, blood was sampled for laboratory investigations, including white blood cell count, platelets, MPV, ESR, and CRP. Peripheral venous blood samples were collected by antecubital venipuncture into Vacutainer tubes (Becton Dickinson™, Rutherford, NJ) containing tripotassium EDTA. Complete blood count (CBC) studies were done within one hour after the blood samples were drawn to minimize changes in platelet size. We also followed a standardized protocol. CBC analysis was performed using Advia 2120i automated analyzer (Siemens Healthcare Diagnostics, Deerfield, IL, USA). CRP levels were measured by the latex-enhanced turbidimetric assay method using a Hitachi 7600 P module (Hitachi, Japan). ESR levels were measured by the TEST 1 (Alifax, Padova, Italy). Strict quality control procedures were adopted.

Radiologic studies such as renal ultrasonography (USG), DMSA scan, and voiding cystourethrogram (VCUG) were performed in all patients. After the patients were admitted with UTI, DMSA scan was performed within the first 5 days. They were divided into groups with APN and lower UTI according to the positive (focal or multifocal perfusion defects) or negative DMSA scan abnormalities.

Statistical analyses were performed, using the SPSS for Windows version 18.0 (SPSS Inc., Chicago, Illinois, USA) and MedCalc version 15.8 (MedCalc Software, Belgium). The independent and paired t-test was used for continuous variables and expressed as mean ± standard deviation. Chi-square test was used to analyze categorical variables. Correlation analysis was also carried out to determine the relationship between two variables by Spearman correlation. Multiple logistic regression analysis was used to find independent predictive factors for DMSA scan abnormalities, indicating APN. To establish the predictive value of the parameters for diagnosing APN, receiver operating characteristic (ROC) curves were plotted for WBC, ESR, CRP and MPV. All differences were considered significant at a value of p < 0.05.

### Ethics statement

The Institutional Review Board and Research Ethics Committee of Yonsei University Severance Hospital approved this study. We were given exemption from getting informed consents by the IRB because the present study is a retrospective study and personal identifiers were completely removed and the data were analyzed anonymously. Our study was conducted according to the ethical standards laid down in the 1964 Declaration of Helsinki and its later amendments.

## Additional Information

**How to cite this article**: Lee, I. R. *et al.* Mean platelet volume in young children with urinary tract infection. *Sci. Rep.* **5**, 18072; doi: 10.1038/srep18072 (2015).

## Figures and Tables

**Table 1:** Demographic data, laboratory and imaging findings of UTI patients with or without DMSA scan abnormality.

	DMSA scan abnormalities (n = 62)	Normal DMSA scan (n = 56)	*P* value
Age (months)	5.4 ± 4.6	4.1 ± 2.0	0.059
Sex (M:F)	44 : 18	42 : 14	0.623
Duration of fever (days)	2.2 ± 1.1	2.0 ± 1.1	0.352
Laboratory findings			
WBC (/μL)	17,608 ± 5,712	14,185 ± 5,210	0.001
Hb (g/dL)	10.9 ± 1.0	11.2 ± 1.0	0.082
PLT (X10^3^/μL)	482 ± 195	472 ± 139	0.742
ESR (mm/hr)	53.1 ± 35.0	37.0 ± 24.8	0.005
CRP (mg/L)	84.1 ± 62.1	36.7 ± 25.8	<0.001
PDW (%)	44.8 ± 6.7	45.7 ± 6.5	0.451
MPV (fL)	7.4 ± 0.5	7.2 ± 0.5	0.011
Imaging findings			
Abnormal USG	48 (77.4%)	35 (62.5%)	0.076
Abnormal VCUG	22 (35.5%)	7 (12.5%)	0.004

DMSA: dimercaptosuccinic acid, WBC: white blood cell, Hb: hemoglobin, PLT: platelet, ESR: erythrocyte sedimentation rate, CRP: C-reactive protein, PDW: platelet distribution width, MPV: Mean platelet volume, USG: ultrasonography, VCUG: voiding cystourethrogram.

**Table 2:** Multiple logistic regression analysis of laboratory parameters associated with renal cortical defects on 99mTechnetium-dimercaptosuccinic acid (DMSA) scintigraphy.

	Odds ratio	95% CI	*P* value
CRP (mg/L)	1.026	1.013–1.040	< 0.001
MPV (fL)	2.393	1.019–5.620	0.045

CRP: C-reactive protein, MPV: Mean platelet volume.

**Table 3:** Receiver operating characteristic curve of MPV and other inflammatory markers for APN.

Variables	Area under curve	Standard error	*P* value	95% CI
WBC (/μL)	0.670	0.050	0.001	0.573–0.768
ESR (mm/hr)	0.626	0.051	0.018	0.526–0.727
CRP (mg/L)	0.764	0.044	<0.001	0.678–0.850
MPV (fL)	0.641	0.051	0.008	0.541–0.742

APN: Acute pyelonephritis, WBC: white blood cell, ESR: erythrocyte sedimentation rate, CRP: C-reactive protein, MPV: Mean platelet volume.

**Table 4:** Diagnostic accuracy of MPV for the prediction of APN.

Variable	Sensitivity	Specificity	Positive predictive value	Negative predictive value	Positive LR	Negative LR
Tekin *et al.*^29^						
MPV ≥ 8.2	81.4(67–92)	86.3(74–94)	83.3(69–93)	84.6(72–93)	5.93(5–7)	0.22(0.1–0.5)
MPV ≥ 9.0	76.7(61–88)	88.2(76–95)	84.6(69–94)	81.8(69–91)	6.52(5–8)	0.26(0.1–0.7)
MPV ≥ 9.5	53.5(38–69)	94.1(84–99)	88.5(70–97)	70.6(58–81)	9.09(7–12)	0.49(0.2–1.6)
Our study						
MPV ≥ 6.9	85.5(74–93)	33.9(22–48)	12.6(6–22)	95.5(83–100)	1.29(1–1.6)	0.43(0.2–0.9)
MPV ≥ 7.4	45.2(33–58)	82.1(70–91)	21.9(8–43)	93.1(86–97)	2.53(1.4–4.7)	0.67(0.5–0.9)
MPV ≥ 8.3	4.8(1–14)	96.4(88–99.6)	13.1(0.03–70)	90.1(83–95)	1.35(0.2–7.8)	0.99(0.9–1.1)

APN: Acute pyelonephritis, MPV: Mean platelet volume, LR: likelihood ratio.

**Table 5:** Comparison of serial MPV levels after antibiotic therapy in UTI patients.

Duration after antibiotics	Initial MPV (fL)	MPV after antibiotics (fL)	*P* value
1 day (n = 17)	7.14 ± 0.38	7.52 ± 0.58	0.031
2 days (n = 32)	7.43 ± 0.49	7.49 ± 0.51	0.479
3 days (n = 30)	7.23 ± 0.45	7.28 ± 0.54	0.613
4 days (n = 8)	7.64 ± 0.87	7.6 ± 0.73	0.834
5 days (n = 8)	7.55 ± 0.69	7.64 ± 0.66	0.564
≥ 6 days (n = 16)	7.39 ± 0.41	7.13 ± 0.48	0.046

UTI: Urinary tract infection, MPV: Mean platelet volume.
